# Admission to hospital following head injury in England: Incidence and socio-economic associations

**DOI:** 10.1186/1471-2458-5-21

**Published:** 2005-03-04

**Authors:** Alan Tennant

**Affiliations:** 1 Professor of Rehabilitation Studies, Academic Unit of Musculoskeletal and Rehabilitation Medicine, The University of Leeds, UK

## Abstract

**Background:**

Head injury in England is common. Evidence suggests that socio-economic factors may cause variation in incidence, and this variation may affect planning for services to meet the needs of those who have sustained a head injury.

**Methods:**

Socio-economic data were obtained from the UK Office for National Statistics and merged with Hospital Episodes Statistics obtained from the Department of Health. All patients admitted for head injury with ICD-10 codes S00.0–S09.9 during 2001–2 and 2002–3 were included and collated at the level of the extant Health Authorities (HA) for 2002, and Primary Care Trust (PCT) for 2003. Incidence was determined, and cluster analysis and multiple regression analysis were used to look at patterns and associations.

**Results:**

112,718 patients were admitted during 2001–2 giving a hospitalised incidence rate for England of 229 per 100,000. This rate varied across the English HA's ranging from 91–419 per 100,000. The rate remained unchanged for 2002–3 with a similar magnitude of variation across PCT's. Three clusters of HA's were identified from the 2001–2 data; those typical of *London*, those of the *Shire counties*, and those of *Other Urban *authorities. Socio-economic factors were found to account for a high proportion of the variance in incidence for 2001–2. The same pattern emerged for 2002–3 at the PCT level. The use of public transport for travel to work is associated with a decreased incidence and lifestyle indicators, such as the numbers of young unemployed, increase the incidence.

**Conclusion:**

Head injury incidence in England varies by a factor of 4.6 across HA's and PCT's. Planning head injury related services at the local level thus needs to be based on local incidence figures rather than regional or national estimates. Socio-economic factors are shown to be associated with admission, including travel to work patterns and lifestyle indicators, which suggests that incidence is amenable to policy initiatives at the macro level as well as preventive programmes targeted at key groups.

## Background

It has been estimated that 6.6% of those attending A&E in any given year have a head injury [1] and over 100,000 people are admitted as a consequence [2]. This incidence of admission to hospital following a head injury is known to vary considerably from locality to locality [3]. Although some technical issues, such as case identification and inaccurate coding may contribute [3,4], there is also evidence to support that demographic- and social factors such as age, gender, environment and ethnicity cause variation in incidence and outcome [5-12]. For example the adjusted odds ratio for age 15–24 years, and the male sex have been found to be significant independent predictors for medically attended brain injury in the USA [13]. Thus the evidence suggests that there is considerable potential for complex interactions which could elevate or depress incidence rates at the local level to a significant degree.

Yet, within the UK, evidence upon which to base local service planning is scarce, as recognised in the recent report of the Health Committee of the UK House of Commons [14]. Consequently the committee recommended that a way is found 'of improving the methods of data collection on incidence, prevalence and severity of head injury and subsequent disability...' (vii). This paper makes an initial response to that recommendation and reports on the incidence of head injury leading to a hospital admission at the local level in England, and examines the socio-economic associations of any variation shown to be present.

## Methods

Hospital Episodes Statistics (HES) record all episodes of continuous in-patient care in hospitals in England and can be obtained from the UK Department of Health. For the year 2001–2, statistics are available for the 95 Health Authorities (HA) extant at the time, and for 2002–3 for Primary Care Trusts (PCT's). Concerned with incidence of new cases, data in the current study is based upon the postcode of residence of the patient. 'First episode' was chosen as a filter variable (as approximately 10% of patients have more than one episode, that is, fall under the care of more than one consultant during their stay). A second filter identified the relevant primary cause ICD10 codes for head injury (S00.0 to S09.9). There is a long history of debate about the appropriate codes for inclusion in such studies, often surrounding the debate between 'head injury' and 'brain injury' [15]. Much was made of the fact that 'fracture of the facial bones' (ICD-9 code 802) was not indicative of brain injury, but new research has shown that excluding this group will omit many with brain injury [16]. The current study is inclusive of all codes, and so includes 'superficial injuries to the head', which nevertheless required admission to hospital. Using census population data for the same areas as the denominator, admission rates per 100,000 are calculated for all ages, and age-specific rates for 0–15 year olds, 16–74 year olds, and those aged 75 years and over. Rates are thus for admission of residents of England and their respective HA or PCT, in an English hospital. Admissions are defined as the first period of in-patient care under one consultant within one healthcare provider.

The 16–74 year old band was chosen explicitly to match the census key statistics which report on various indicators for the economically active population, deemed to be 16–74 years of age. Census Key Statistics are available from the UK Office of National Statistics and include a range of demographic, social and economic variables that can be presented as percentage indicators. Those chosen for inclusion in the current study are shown in Table 1. The indicator for qualification at level 4/5+ represents those with a first degree, a higher degree, NVQ levels 4 and 5, HNC, HND, qualified teacher, medical doctor, dentist, nurse, midwife or health visitor.

**Table 1:** Key statistics included in the study and presented as percentage indicators.

**Indicator**

% born outside the UK
% with limiting long-standing illness
% permanently sick of working age
% 16–24 who are unemployed
% age 50+ who are unemployed
% Unemployed
% without any qualifications
% with qualifications at grade 4/5+
% using private transport to work
% using public transport to work
% of households without a car
% who own (or buying) their home
% who rent home privately
% living in overcrowded homes
% lone parent families

In addition, the Townsend Deprivation Index is calculated using the percentage of households with no car, not owner occupied, overcrowded and those economically active who are unemployed [17]. The latter two are transformed logarithmically, and then each variable is transformed to a normal distribution using the means and standard deviations for England as a whole. The variables are summed giving an average for England of zero, with negative values indicating less deprived areas, positive values more deprived areas.

The UK Census key statistics are currently made available at the Primary Care Trust (PCT) level, and thus it was necessary to aggregate these data up to the HA level to match the 2001–2 admission data which was provided at the Health Authority level. The majority of PCT's fit neatly into the 95 Health Authority areas extant in 2002. However, there are some slight variations such that aggregated populations of the HA derived from their respective PCT's match exactly the independent figures (provided separately from the ONS) of 2002 HA populations in 90% of cases, and differ by up to 5% for the rest. For example, a small number of Enumeration Districts (the lowest level census tract) belonging to the Preston PCT, which belongs in North West Lancashire HA, are allocated to East Lancashire. No attempt was made to adjust for these slight variations in boundaries. Key statistic indicators for 2001–2 were thus derived from the aggregated PCT data, based on their aggregated population which, for a few HA's, will differ slightly from the population used to estimate the admission rates for head injury. There was a one-to-one relationship between admission data and census data at the PCT level for the 2002–3 data.

Patterns of head injury and socio-economic indicators are determined by Two-Step Cluster Analysis. The procedure is an exploratory tool designed to reveal natural groupings (or clusters) within a data set that would otherwise not be apparent. The association between the various socio-economic factors, and the overall rate for head injury is explored through a multiple regression model using the 2001–2 data at the HA level. This model is validated on the 2002–3 data at the PCT level.

## Funding and ethics

The project was funded by the UK Department of Health. The author is independent of the funding body. Ethical approval was not required.

## Results

A total of 112,718 admissions were recorded for 2001–2. The gives an incidence rate for admission to hospital following a head injury in England in 2001–2, for all ages, of 229.4 per 100,000 (Table 2). Of these, 31.2% were aged 0–15; 56.2% were aged 16–74 and 12.6% aged 75 years and over. The total incidence varies by a factor of 4.6 from 90.7 per 100,000 in Brent and Harrow, to 419.4 in Liverpool. Rates for children and the elderly are much higher than for those aged 16–74 years. The highest incidence rate for children was East Lancashire at 637.7 per 100, 000 aged 0–15; the highest for the elderly was North Cheshire at 799.8 per 100,000 aged 75 years and over (See Additional File 1). The incidence for 2002–3 was identical at 229.1 per 100,000 all ages (Table 3). While the magnitude of difference for the overall all-age incidence remained the same at 4.6, at the PCT level the age-specific variability appears greater, with the highest rate for children being 881.7 per 100,000 (North Manchester, see Additional File 2) and that for those aged 75 years and over, 1116.0 per 100,000 in Central Liverpool.

**Table 2:** Hospitalised Incidence rate for head injury in England in 2001–2. Estimated rate per 100,000; for those aged 0–15; 16–74; and 75 years and over, and in total. Estimates for England, and the highest and lowest incidence for health authorities.

**Health Authority**	**RATE 0–15**	**RATE 16–74**	**RATE 75+**	**Total RATE**
England	355.8	178.1	383.8	229.4

Liverpool	465.9	376.9	741.5	419.4
Tees	621.7	346.9	514.5	416.6
North Cheshire	488.0	356.0	799.8	411.5
East Lancashire	637.7	298.5	638.3	399.9
Sunderland	461.4	344.2	609.9	385.0

South Essex	235.0	116.4	223.2	148.9
Barking and Havering	223.9	117.4	210.0	147.4
North Essex	256.0	103.0	268.1	146.8
Bexley, Bromley & Greenwich	218.1	105.4	181.7	134.4
Brent and Harrow	119.5	81.7	106.7	90.7

**Table 3:** Hospitalised Incidence rate for head injury in England in 2002–3. Estimated rate per 100,000; for those aged 0–15; 16–74; and 75 years and over, and in total. Estimates for England, and the highest and lowest incidence for PCT's.

**PCT**	**RATE 0–15**	**RATE 16–74**	**RATE 75+**	**Total RATE**
ENGLAND	339.0	179.5	410.8	229.1

Preston	577.4	478.3	611.9	508.0
Central Liverpool	516.5	429.0	1116.0	488.6
Central Derby	698.0	362.9	895.5	479.0
Middlesbrough	582.4	431.1	550.6	472.5
Birkenhead and Wallasey	473.0	436.3	777.3	471.0

Colchester	175.5	95.4	195.6	118.5
Cherwell Vale	193.7	88.9	174.9	117.1
Brent	134.2	108.7	95.4	113.1
South West Oxfordshire	153.2	98.5	136.2	112.5
Harrow	124.7	97.0	198.5	109.8

Taking the overall incidence rate for 2001–2, together with the key statistics in Table 1, a Cluster Analysis indicated three clear groupings of Health Authorities in England (Table 4). The first can be described as 'London', and includes those authorities located within London. Here, 26.7 % of the population was born outside of the UK and the workforce is better qualified than elsewhere. A lower than average number of people own their own homes, and more people travel to work by public transport than by their own car. It is more overcrowded which contributes to a high deprivation index at 5.5. The admission rate is lower than average at 176 per 100,000 all ages.

**Table 4:** Cluster analysis of socio-economic indicators associated with head injury in England.

	**Cluster**			
**Characteristic**	**London**	**Shire**	**Other Urban**	**England**
				
Admission Rate – all ages	176	206	288	229
				
% Born out of UK	26.7	5.7	4.8	9.2
% of working age permanently sick	4.5	4.2	7.6	5.7
% age 16–24 unemployed	5.5	4.6	7.4	5.9
% without qualifications	22.4	26.7	35.1	29.7
% qualified at level 5+	33.2	19.6	14.8	19.3
% using private transport to work	35.2	67.0	65.3	62.2
% using public transport to work	43.8	8.9	14.2	15.6
% households without a car	38.3	20.1	32.3	27.6
% owning (or buying) own home	55.8	74.0	66.5	68.6
% Lone parent household	10.9	8.2	10.9	9.4
% living in overcrowded homes	18.0	5.1	5.7	7.0
Average Townsend Index	5.5	-2.7	1.2	0.0

The second cluster can be described as the 'Shire' counties, typical of the more rural areas found within England, including Cambridgeshire and Worcestershire. They are characterised by a large proportion of people owning their own home, travelling to work in their own car, and with few of the population born outside of the UK. Although a quarter of the population are without qualifications, the deprivation Index is low at -2.7. The average incidence admission rate for this cluster is 206 per 100,000 all ages.

The third cluster can be described as 'Other Urban', and typical of all the Midland and Northern cities of England. They are characterised by a higher proportion of people with limited long standing illness; those of working age who are permanently off sick; of those without qualifications and of those aged 16–24 who are unemployed. The deprivation Index is above average at 1.2, and the admission rate is high at 288 per 100,000 all ages.

**Figure 1:**
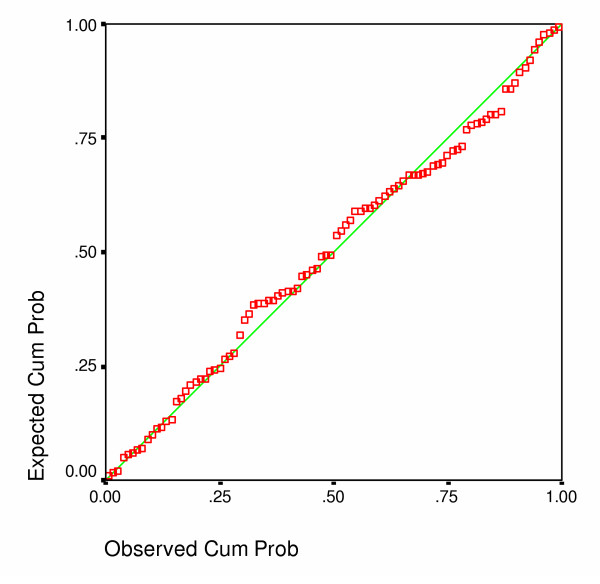
Normal probability plot of regression with Rate per 100,000 as dependent variable.

A regression analysis with overall rate as the dependent variable shows how these various indicators come together to predict incidence (Table 5). For every one percent increase in the 16–24 unemployment rate, the hospital admission rate for head injury increases by 17.4 per 100,000 all ages. For every one percent increase in those permanently sick of working age, the rate increases by 16.0 per 100,000 all ages. For every one percent increase in lone parent families the rate increase by 11.0 per 100,000 all ages. In contrast for every one percent increase in the use of public transport to go to work, the rate decreases by 4.6 per 100,000 all ages. With an adjusted R^2 ^of 0.698, the model explains a large proportion of the variation in admission rates for a head injury (F 36.77; p < 0.01; and with an acceptable pattern of residuals [Figure 1]). The most important variable (highest beta) was use of public transport to work. These significant predictors were then entered into another regression model, along with all possible two-way interactions. The latter were entered in a stepwise fashion, but none gained statistical significance.

**Table 5:** Indicators of admission to hospital with a head injury: All ages 2001–2. β is increase (decrease) in rate per 100,000 admissions for each % increase of indicator.

**Indicator**	**β**	**95% CI for β**
% Unemployed within age of 16–24	17.431	5.707 to 29.154
% Permanently sick of working age	16.019	6.226 to 25.773
% Lone parent households	11.012	2.892 to 19.132
% Using public transport for work	-4.563	-6.204 to -2.923
% Without qualifications	-7.781	-10.741 to -4.821

The model was then validated upon the 2002–3 data at the PCT level (Table 6). A similar model emerged with adjusted R^2 ^of 0.579, again explaining a large proportion of the variation in admission at the PCT level (F70.078; p < 0.01). On this occasion the proportion of households without a car was added to the previous set of predictors. Once again, the proportion travelling to work by public transport was the most important variable.

**Table 6:** Indicators of admission to hospital with a head injury: All ages 2002–3. β is increase (decrease) in rate per 100,000 admissions for each % increase of indicator.

**Indicator**	**β**	**95% CI for β**
% Unemployed within age of 16–24	7.171	0.630 to 13.711
% Permanently sick of working age	10.772	4.900 to 16.645
% Lone parent households	11.034	6.018 to 16.051
% Using public transport for work	-4.412	-5.396 to -3.427
% Without qualifications	-5.214	-7.032 to -3.396
% No car	4.359	2.982 to 5.735

## Discussion

Head injury is common; there were 112,718 recorded admissions for English residents during the year April 2001 to March 2002, giving a hospitalised incidence rate of 229.4 per 100,000 all ages. Remaining the same for 2002–3 this incidence is similar to that of stroke although the latter is experienced in a predominately older population [18,19]. However, head injury affects a predominately younger population and carries with it a high potential economic impact. For example, based on these latest incidence figures and published evidence from other studies, we can estimate that about 4700 of those admitted in any given year, and who are considered to be economically active (aged 16–74) and in employment at the time of their injury would be unable to return to their work at 6 weeks [20,21].

Admission rates for England were found to vary by a factor of 4.6 between both health authorities and PCT's. The rates may slightly underestimate the true incidence as those residents of England treated elsewhere are not included. Also, under-reporting may depress incidence to an unknown extent [4,22] and, if underreporting varies by locality, will further contribute to variation. Standardised admission policies for those presenting with a head injury were not agreed at the time that these data were collected and this may also contribute to variation in incidence. The publication of the NICE guidelines for the early management of head injury may reduce any variation attributable to differing admission policies [23].

Half of all health authorities in England, and slightly more PCT's (55%) had an incidence rate which fell below or above the England average by at least 20%. This has important implications for planning local services in response to head injury. Clearly using the overall England incidence rate is unlikely to be helpful. This variation also causes problems in extrapolating the results from research undertaken in a single locality. Estimates of the number of people disabled by head injury, presented as a rate (e.g. 150 per 100000), may have little relation to reality at the local level. The disability rate is conditional upon the local incidence where the study was undertaken. Consequently it is important that research findings should be presented as proportions of the local base incidence if they are to be of any use for planning elsewhere. The extrapolations given above on return to work are based on this approach. Nevertheless such extrapolation assumes equal case mix (e.g. similar mechanisms of injury) which is also unlikely. Case mix will, to a certain extent, be a reflection of local socio-economic factors, particularly associated with sub-populations displaying chronic conditions, including alcohol misuse, shown to have significant impact on outcome [20,21,24,25].

The extent of deprivation has also previously been associated with higher incidence of head injury as well as general workload for primary care [26,27]. The data presented here tell a slightly different story. Although Camden and East London health authorities are above the English average for incidence, and have the highest levels of deprivation, and likewise Tower Hamlets and City and Hackney PCT's, the overall correlation between deprivation and incidence is low (0.21). It is the rate of 16–24 unemployment that contributes to higher incidence, and those of working age who report themselves as permanently unable to work because of ill health. Set against these 'lifestyle factors', other factors mediate the incidence level. Thus London, of all cities in England, with the highest localities of deprivation, but with its extensive public transport system, displays a lower overall incidence rate. These findings are consistent for both years examined. However, it is unclear if, at all, reductions in the number of Accident and Emergency beds in the capital (generally from a higher base than elsewhere in the country), and the consequent reduced capacity to admit and observe, may also have contributed to this lower incidence. In contrast, the very high incidence of admission for children who are resident in the area of North Manchester PCT may be associated with the accessibility to the children's hospital located in that area. Indeed, local service and residential patterns, for example, large residential or nursing homes located with a PCT's boundary, may have significant impact on incidence (as expressed by place of residence) at the very local level.

Where public transport is not used for journeys to work, incidence is much higher and combined with higher unemployment rates, and other significant indicators associated with 'lifestyle indicators', high incidence rates are observed in the cities and other urban communities in the Midlands and Northern England, including Teeside and Mersyside. As demographic, social and economic factors appear to account for half the variation observed in hospitalised admission rates, one implication is that incidence can be modified at the macro policy level. For example, *ceteris paribus*, we might expect to see the London incidence fall as a consequence of congestion charging and associated increase in the use of public transport. Policies targeted at reducing unemployment amongst the 16–24 year old age group may also be expected to reduce incidence. Also, a continuing emphasis on prevention is clearly needed, both for the young and the old, to reduce their very high incidence rates.

Some technical limitations are worth mentioning. The likelihood distance measure used in the two-step cluster analysis assumes that variables in the cluster model are independent. Further, each continuous variable is assumed to have a normal (Gaussian) distribution, although in practice the technique is robust to violation of these assumptions. In fact, reassuringly, the results of the linear regression model do suggest independent main effects for several of the key variables and, importantly, the absence of interaction effects. Also it is unknown how, if at all, the slight variation in population denominators used to calculate incidence and the socio-economic indicators for the 2001–2 data could influence these results. However, replication upon the 2002–3 data at the PCT level suggests a robust model. Due to censoring of data of elderly admissions at the PCT level we were not able to confidently include the proportion of females admitted in our validation model, and thus we do not know if variation in male-female ratios at the local level was also a contributing factor to variance in incidence.

It is also worth restating that the accuracy of case ascertainment, and the coding of head injuries upon admission to hospital is known to underestimate the true incidence [4,22] but how much this varies across localities, and its consequent potential to bias these results, is unknown. Finally, it is possible that a few patients are double counted in that those transferred will be seen as a new admission into a different hospital, thus leading to an unknown over-estimate of incidence. However, these are likely to be those with the most severe injuries admitted initially to hospitals without neurosurgery, and numbers are likely to be small.

## Conclusion

Incidence of head injury in England is high, similar to stroke if just admissions are considered, and the data show considerable variability at the local level. Given that most of the estimates of the potential impact of head injury rely on studies undertaken in a single locality [21,28,29] those planning for rehabilitation and other services must take care to identify the proportion of those with a head injury that experience the sequelae under scrutiny (e.g disability; job loss), together with the case mix, and not just rely on headline rates which are conditional upon local incidence.

To assist in planning for services, data at the local level can now readily be obtained from on-line data sets from the Department of Health. At the national level they can be downloaded on-line at:



While we have as yet little understanding of how case mix is related to, or interacts with socio-economic factors to mediate medium or longer term-outcomes, the association of such factors with incidence suggests that economic and social policies, for example in the development of prevention programmes, and in encouraging the use of public transport for journeys to work, may have a significant impact on reducing the incidence of head injury.

## Competing interests

The author(s) declare that they have no competing interests.

## Pre-publication history

The pre-publication history for this paper can be accessed here:



## Supplementary Material

Additional File 1 Hospitalised Incidence rate for head injury in England in 2001–2. Estimated rate per 100,000; for those aged 0–15; 16–74; and 75 years and over, and in total. Estimates for England, and extant Regions and Health Authorities at the time. Click here for file

Additional File 2 Incidence of admission to hospital for head injury for England, and each Primary Care Trust (PCT) in England 2002–3; Estimated rate per 100,000; for those aged 0–15; 16–74; and 75 years and over, and in total. Estimates for England, and PCT's. Click here for file
