# Supplementary material for: Admission to hospital following head injury in England: Incidence and socio-economic associations
Source: BMC Public Health. 2005 Mar 4;5:21. doi: 10.1186/1471-2458-5-21 (PMC554988; doi:10.1186/1471-2458-5-21)
Supplement: Additional File 1 — Hospitalised Incidence rate for head injury in England in 2001–2. Estimated rate per 100,000; for those aged 0–15; 16–74; and 75 years and over, and in total. Estimates for England, and extant Regions and Health Authorities at the time. [file 1471-2458-5-21-S1.doc]

**Additional File 1. Hospitalised Incidence rate for head injury in England in 2001-2. Estimated rate per 100,000; for those aged 0-15; 16-74; and 75 years and over, and in total. Estimates for England, and extant Regions and Health Authorities at the time.**

| **Region &** Health Authority | **RATE 0-15** | **RATE 16-74** | **RATE 75+** | **Total RATE** |
| --- | --- | --- | --- | --- |
| England | 355.81 | 178.14 | 383.76 | 229.39 |
| **Northern and Yorkshire** | 413.77 | 232.63 | 409.61 | 282.35 |
| Bradford | 381.24 | 222.64 | 402.41 | 271.77 |
| Calderdale and Kirklees | 342.50 | 206.70 | 323.87 | 244.50 |
| County Durham | 477.77 | 206.82 | 337.28 | 268.90 |
| East Riding | 236.04 | 206.36 | 374.21 | 225.40 |
| Gateshead and South Tyneside | 515.81 | 275.61 | 436.84 | 335.53 |
| Leeds | 386.92 | 229.29 | 571.06 | 285.71 |
| Newcastle and North Tyneside | 493.00 | 217.67 | 359.78 | 281.23 |
| North Cumbria | 457.70 | 263.71 | 375.58 | 309.27 |
| Northumberland | 345.36 | 218.71 | 446.92 | 260.75 |
| North Yorkshire | 280.09 | 179.05 | 362.29 | 213.77 |
| Sunderland | 461.36 | 344.15 | 609.86 | 384.96 |
| Tees | 621.75 | 346.90 | 514.54 | 416.62 |
| Wakefield | 550.40 | 186.35 | 260.60 | 265.89 |
| **Trent** | 424.25 | 198.03 | 408.42 | 259.34 |
| Barnsley | 451.92 | 220.12 | 507.33 | 288.45 |
| Doncaster | 572.70 | 217.52 | 388.30 | 303.63 |
| Leicestershire | 387.61 | 179.44 | 370.54 | 235.37 |
| Lincolnshire | 374.76 | 205.46 | 427.81 | 257.17 |
| North Derbyshire | 307.11 | 151.56 | 286.66 | 192.59 |
| North Nottinghamshire | 402.42 | 230.12 | 480.16 | 283.87 |
| Nottingham | 548.39 | 204.84 | 413.01 | 287.30 |
| Rotherham | 483.15 | 232.38 | 572.63 | 309.05 |
| Sheffield | 339.36 | 178.63 | 415.84 | 228.55 |
| Southern Derbyshire | 456.55 | 185.87 | 376.82 | 255.46 |
| South Humber | 416.17 | 232.46 | 397.07 | 283.76 |
| **Eastern** | 299.30 | 138.33 | 340.66 | 186.41 |
| Bedfordshire | 225.43 | 121.94 | 301.79 | 155.31 |
| Cambridgeshire | 382.33 | 188.19 | 530.19 | 250.59 |
| Hertfordshire | 325.97 | 135.64 | 338.12 | 189.37 |
| Norfolk | 360.65 | 151.79 | 290.49 | 202.95 |
| North Essex | 256.02 | 103.04 | 268.08 | 146.75 |
| South Essex | 234.99 | 116.37 | 223.16 | 148.90 |
| Suffolk | 298.34 | 158.02 | 472.04 | 213.90 |
| **London** | 251.03 | 144.29 | 361.05 | 178.65 |
| Barking and Havering | 223.93 | 117.41 | 209.99 | 147.35 |
| Barnet, Enfield & Haringey | 184.24 | 128.78 | 412.35 | 157.84 |
| Bexley, Bromley & Greenwich | 218.05 | 105.35 | 181.67 | 134.41 |
| Brent and Harrow | 119.47 | 81.73 | 106.68 | 90.69 |
| Camden and Islington | 292.97 | 196.87 | 748.52 | 240.23 |
| Croydon | 251.50 | 129.28 | 288.89 | 165.72 |
| Ealing, Hammersmith and Hounslow | 204.41 | 166.20 | 390.37 | 185.40 |
| East London and The City | 364.82 | 188.20 | 455.98 | 241.74 |
| Hillingdon | 266.05 | 155.35 | 510.88 | 202.12 |
| Kensington & Chelsea and Westminster | 166.21 | 131.15 | 388.15 | 151.09 |
| Kingston and Richmond | 256.43 | 109.53 | 418.13 | 159.25 |
| Lambeth, Southwark and Lewisham | 292.86 | 209.25 | 577.02 | 243.57 |
| Merton, Sutton and Wandsworth | 390.28 | 123.02 | 273.42 | 181.86 |
| Redbridge and Waltham Forest | 215.76 | 138.94 | 396.43 | 171.59 |
| **South East** | 328.64 | 138.99 | 376.20 | 195.64 |
| Berkshire | 25.78 | 122.83 | 264.99 | 157.60 |
| Buckinghamshire | 340.96 | 134.93 | 274.76 | 187.58 |
| East Kent | 300.36 | 146.18 | 402.09 | 201.69 |
| East Surrey | 355.75 | 108.64 | 345.72 | 177.69 |
| East Sussex, Brighton and Hove | 403.20 | 155.01 | 435.47 | 230.63 |
| Isle of Wight, Portsmouth & SE Hants | 383.20 | 217.21 | 733.94 | 294.57 |
| North and Mid Hampshire | 352.56 | 136.36 | 322.15 | 192.78 |
| Northamptonshire | 298.39 | 126.89 | 310.13 | 175.33 |
| Oxfordshire | 238.14 | 145.80 | 315.10 | 175.72 |
| Southampton and South West Hants | 357.38 | 137.51 | 308.97 | 194.27 |
| West Kent | 348.30 | 133.91 | 431.86 | 199.46 |
| West Surrey | 385.49 | 108.71 | 275.92 | 174.44 |
| West Sussex | 279.70 | 126.34 | 309.70 | 174.76 |
| **South West** | 330.91 | 163.61 | 394.80 | 216.76 |
| Avon | 295.26 | 129.33 | 329.66 | 177.36 |
| Cornwall and Isles of Scilly | 508.21 | 281.65 | 612.78 | 355.70 |
| Dorset | 314.87 | 158.03 | 287.03 | 200.51 |
| Gloucestershire | 335.10 | 168.83 | 415.24 | 222.65 |
| North and East Devon | 357.17 | 165.46 | 420.53 | 226.24 |
| Somerset | 314.29 | 133.60 | 373.57 | 191.53 |
| South and West Devon | 336.22 | 187.63 | 493.83 | 245.48 |
| Wiltshire | 256.65 | 124.05 | 305.88 | 164.75 |
| **West Midlands** | 358.25 | 160.30 | 315.67 | 212.93 |
| Birmingham | 448.02 | 204.99 | 445.06 | 278.79 |
| Coventry | 279.35 | 183.75 | 272.93 | 210.74 |
| Dudley | 340.31 | 121.61 | 269.72 | 175.97 |
| Herefordshire | 316.43 | 199.07 | 489.15 | 248.22 |
| North Staffordshire | 280.23 | 132.76 | 130.19 | 160.99 |
| Sandwell | 375.18 | 172.60 | 368.43 | 231.53 |
| Shropshire | 445.08 | 193.46 | 355.86 | 256.38 |
| Solihull | 387.43 | 114.22 | 307.95 | 185.95 |
| South Staffordshire | 263.34 | 120.09 | 250.24 | 157.57 |
| Walsall | 459.98 | 196.22 | 297.79 | 260.35 |
| Warwickshire | 303.77 | 178.37 | 456.66 | 223.77 |
| Wolverhampton | 324.12 | 149.33 | 97.51 | 181.76 |
| Worcestershire | 325.30 | 102.36 | 224.69 | 155.14 |
| **North West** | 459.43 | 259.72 | 452.88 | 315.29 |
| Bury and Rochdale | 500.58 | 249.62 | 548.13 | 325.00 |
| East Lancashire | 637.74 | 298.52 | 638.33 | 399.91 |
| Liverpool | 465.92 | 376.94 | 741.52 | 419.36 |
| Manchester | 577.73 | 257.23 | 203.70 | 322.03 |
| Morecambe Bay | 436.35 | 207.06 | 431.48 | 270.28 |
| North Cheshire | 488.04 | 355.98 | 799.79 | 411.25 |
| North West Lancashire | 382.52 | 286.50 | 408.43 | 316.13 |
| Salford and Trafford | 432.61 | 227.49 | 335.72 | 277.30 |
| Sefton | 349.59 | 262.68 | 450.36 | 296.51 |
| South Cheshire | 358.11 | 167.82 | 346.87 | 218.92 |
| South Lancashire | 431.45 | 225.41 | 419.91 | 279.83 |
| St Helens and Knowsley | 425.39 | 331.49 | 566.50 | 366.62 |
| Stockport | 431.56 | 167.66 | 342.37 | 234.41 |
| West Pennine | 527.33 | 290.11 | 420.64 | 351.29 |
| Wigan and Bolton | 420.54 | 192.35 | 308.21 | 248.02 |
| Wirral | 403.25 | 326.21 | 493.75 | 356.40 |
