# Supplementary material for: Admission to hospital following head injury in England: Incidence and socio-economic associations
Source: BMC Public Health. 2005 Mar 4;5:21. doi: 10.1186/1471-2458-5-21 (PMC554988; doi:10.1186/1471-2458-5-21)
Supplement: Additional File 2 — Incidence of admission to hospital for head injury for England, and each Primary Care Trust (PCT) in England 2002–3; Estimated rate per 100,000; for those aged 0–15; 16–74; and 75 years and over, and in total. Estimates for England, and PCT's. [file 1471-2458-5-21-S2.doc]

**Additional File 2. Incidence of admission to hospital for head injury for England, and each Primary Care Trust in England 2002-3; Estimated rate per 100,000; for those aged 0-15; 16-74; and 75 years and over, and in total**

| PCT | Under 16 | 16-74 | 75+ | All Ages |
| --- | --- | --- | --- | --- |
| ENGLAND | 338.96 | 179.49 | 410.81 | 229.07 |
| Adur, Arun and Worthing | 303.86 | 161.16 | 370.40 | 214.70 |
| Airedale | 403.71 | 191.25 | 344.18 | 248.56 |
| Amber Valley | 260.14 | 158.84 | 328.67 | 192.32 |
| Ashfield | 351.66 | 222.22 | 272.48 | 252.19 |
| Ashford | 281.46 | 148.67 | 506.20 | 204.46 |
| Ashton, Leigh and Wigan | 351.13 | 194.83 | 355.34 | 237.21 |
| Barking and Dagenham | 271.16 | 152.32 | 283.07 | 189.70 |
| Barnet | 177.24 | 105.21 | 374.70 | 139.24 |
| Barnsley | 478.21 | 261.47 | 507.36 | 323.76 |
| Basildon | 305.93 | 147.00 | 188.53 | 185.23 |
| Bassetlaw | 377.04 | 218.32 | 430.11 | 265.52 |
| Bath and North East Somerset | 301.80 | 124.22 | 318.77 | 173.92 |
| Bebington and West Wirral | 343.02 | 222.01 | 558.94 | 277.93 |
| Bedford | 205.32 | 125.35 | 416.26 | 162.26 |
| Bedfordshire Heartlands | 213.18 | 112.17 | 255.57 | 142.51 |
| Bexhill and Rother | 422.96 | 138.78 | 610.94 | 259.72 |
| Bexley | 228.73 | 96.05 | 234.15 | 134.21 |
| Billericay; Brentwood and Wickford | 168.39 | 100.79 | 213.34 | 122.64 |
| Birkenhead and Wallasey | 473.00 | 436.34 | 777.27 | 470.95 |
| Blackburn with Darwen | 513.22 | 353.05 | 732.43 | 416.82 |
| Blackpool | 387.02 | 242.67 | 462.39 | 290.97 |
| Blackwater Valley and Hart | 235.11 | 149.01 | 339.05 | 177.66 |
| Bolton | 363.01 | 214.81 | 463.79 | 264.71 |
| Bournemouth | 365.08 | 187.96 | 262.43 | 227.32 |
| Bracknell Forest | 290.69 | 106.56 | 202.91 | 152.35 |
| Bradford City | 368.52 | 211.02 | 471.94 | 268.24 |
| Bradford South and West | 400.04 | 198.46 | 442.33 | 259.93 |
| Brent | 134.18 | 108.70 | 95.37 | 113.11 |
| Brighton and Hove City | 269.02 | 145.84 | 564.76 | 202.57 |
| Bristol North | 296.11 | 126.64 | 286.45 | 173.66 |
| Bristol South and West | 284.73 | 113.21 | 361.13 | 160.94 |
| Broadland | 334.68 | 93.83 | 299.68 | 156.10 |
| Bromley | 207.23 | 129.02 | 461.04 | 171.89 |
| Broxtowe & Hucknall | 419.05 | 162.68 | 450.36 | 233.11 |
| Burnley, Pendle and Rossendale | 603.50 | 258.73 | 468.76 | 351.82 |
| Burntwood, Lichfield and Tamworth | 251.69 | 97.09 | 438.31 | 149.85 |
| Bury | 395.34 | 297.69 | 600.45 | 339.41 |
| Calderdale | 380.56 | 214.29 | 258.82 | 253.11 |
| Cambridge City | 299.59 | 280.04 | 693.70 | 311.40 |
| Camden | 225.16 | 242.97 | 840.93 | 270.17 |
| Cannock Chase | 356.02 | 92.73 | 146.92 | 151.24 |
| Canterbury and Coastal | 416.65 | 154.03 | 352.18 | 222.65 |
| Carlisle and District | 536.52 | 260.54 | 369.51 | 321.94 |
| Castle Point and Rochford | 211.04 | 89.22 | 200.79 | 121.75 |
| Central Cheshire | 306.58 | 163.66 | 438.94 | 212.75 |
| Central Cornwall | 434.03 | 332.75 | 590.95 | 377.19 |
| Central Derby | 697.99 | 362.86 | 895.52 | 478.95 |
| Central Liverpool | 516.51 | 429.04 | 1115.96 | 488.55 |
| Central Manchester | 425.59 | 225.38 | 218.40 | 265.95 |
| Central Suffolk | 208.38 | 119.06 | 280.41 | 150.83 |
| Charnwood and North West Leicest’ | 299.22 | 149.72 | 346.63 | 192.56 |
| Chelmsford | 245.93 | 120.36 | 359.60 | 162.18 |
| Cheltenham and Tewkesbury | 365.25 | 195.40 | 400.65 | 245.44 |
| Cherwell Vale | 193.69 | 88.94 | 174.91 | 117.12 |
| Cheshire West | 330.53 | 119.92 | 104.34 | 157.40 |
| Chesterfield | 352.24 | 197.78 | 468.71 | 250.90 |
| Chiltern and South Bucks | 286.19 | 126.21 | 392.45 | 179.15 |
| Chingford, Wanstead and Woodford | 195.76 | 106.31 | 546.71 | 159.92 |
| Chorley and South Ribble | 413.53 | 336.19 | 874.91 | 388.12 |
| City and Hackney | 351.34 | 245.86 | 334.22 | 273.8 |
| Colchester | 175.45 | 95.42 | 195.57 | 118.45 |
| Cotswold and Vale | 234.63 | 145.30 | 459.27 | 191.74 |
| Coventry | 279.55 | 197.83 | 228.28 | 217.39 |
| Craven, Harrogate and Rural District | 272.13 | 224.60 | 476.29 | 256.15 |
| Crawley | 391.20 | 133.51 | 120.81 | 187.48 |
| Croydon | 234.65 | 125.17 | 244.24 | 156.39 |
| Dacorum | 345.84 | 97.78 | 278.95 | 162.56 |
| Darlington | 575.90 | 242.57 | 540.13 | 334.23 |
| Dartford, Gravesham and Swanley | 253.47 | 109.35 | 402.58 | 159.46 |
| Daventry and South Northamptonshire | 449.59 | 128.47 | 298.01 | 207.37 |
| Derbyshire Dales and South Derbyshire | 270.72 | 134.91 | 565.19 | 192.64 |
| Derwentside | 612.56 | 318.32 | 480.91 | 387.9 |
| Doncaster Central | 528.84 | 293.52 | 545.89 | 363.68 |
| Doncaster East | 467.04 | 187.78 | 337.88 | 254.63 |
| Doncaster West | 542.07 | 253.53 | 459.20 | 330.63 |
| Dudley Beacon and Castle | 255.38 | 130.68 | 295.68 | 168.81 |
| Dudley South | 241.05 | 111.76 | 269.30 | 148.55 |
| Durham and Chester-le-Street | 603.31 | 239.86 | 437.49 | 316.12 |
| Durham Dales | 311.49 | 127.29 | 326.13 | 179.50 |
| Ealing | 187.46 | 175.28 | 432.35 | 191.73 |
| Easington | 512.95 | 314.48 | 266.00 | 352.15 |
| East Cambridgeshire and Fenland | 355.18 | 176.74 | 606.06 | 247.33 |
| East Devon | 273.99 | 113.76 | 537.82 | 201.35 |
| East Elmbridge and Mid Surrey | 347.91 | 109.46 | 370.23 | 179.83 |
| East Hampshire | 353.39 | 189.96 | 749.14 | 269.41 |
| East Kent Coastal | 368.80 | 202.50 | 401.86 | 256.84 |
| East Leeds | 401.67 | 229.57 | 660.94 | 299.31 |
| East Lincolnshire | 369.32 | 196.61 | 409.68 | 248.34 |
| East Staffordshire | 331.86 | 140.68 | 380.42 | 197.82 |
| East Surrey | 385.31 | 114.84 | 324.26 | 185.57 |
| East Yorkshire | 228.61 | 120.43 | 380.90 | 161.81 |
| Eastbourne Downs | 377.53 | 172.01 | 420.66 | 243.83 |
| Eastern Birmingham | 499.86 | 213.77 | 639.73 | 315.48 |
| Eastern Cheshire | 339.58 | 173.48 | 349.87 | 219.67 |
| Eastern Hull | 357.93 | 274.36 | 601.31 | 315.31 |
| Eastern Leicester | 321.23 | 208.18 | 461.87 | 248.84 |
| Eastern Wakefield | 534.85 | 196.27 | 213.97 | 269.18 |
| Eastleigh and Test Valley South | 328.05 | 100.69 | 263.02 | 159.81 |
| Eden Valley | 346.27 | 167.70 | 529.28 | 231.38 |
| Ellesmere Port and Neston | 444.76 | 159.67 | 175.10 | 220.39 |
| Enfield | 166.11 | 121.90 | 332.23 | 145.12 |
| Epping Forest | 302.85 | 98.57 | 273.56 | 153.02 |
| Erewash | 381.48 | 195.47 | 544.79 | 259.77 |
| Exeter | 513.84 | 201.95 | 324.95 | 266.1 |
| Fareham and Gosport | 301.06 | 158.58 | 815.71 | 238.62 |
| Fylde | 343.05 | 147.76 | 434.73 | 214.43 |
| Gateshead | 519.80 | 240.62 | 535.24 | 317.03 |
| Gedling | 404.67 | 173.76 | 294.95 | 227.22 |
| Great Yarmouth | 247.68 | 188.25 | 370.33 | 216.94 |
| Greater Derby | 368.85 | 231.98 | 452.93 | 276.91 |
| Greenwich | 259.45 | 135.90 | 200.43 | 166.98 |
| Guildford and Waverley | 491.94 | 121.29 | 370.85 | 211.06 |
| Halton | 464.05 | 330.43 | 592.57 | 374.76 |
| Hambleton and Richmondshire | 467.16 | 233.02 | 592.80 | 305.46 |
| Hammersmith and Fulham | 426.06 | 224.98 | 729.57 | 283.22 |
| Haringey | 195.05 | 150.58 | 456.42 | 172.74 |
| Harlow | 526.56 | 156.09 | 288.78 | 243.75 |
| Harrow | 124.73 | 97.00 | 198.51 | 109.76 |
| Hartlepool | 477.35 | 267.32 | 130.74 | 303.57 |
| Hastings and St Leonards | 579.16 | 257.37 | 361.96 | 335.18 |
| Havering | 218.22 | 111.58 | 265.22 | 145.37 |
| Heart of Birmingham Teaching | 408.29 | 253.63 | 733.50 | 317.47 |
| Herefordshire | 322.73 | 163.43 | 344.55 | 211.01 |
| Hertsmere | 270.33 | 91.11 | 443.32 | 157.76 |
| Heywood and Middleton | 610.35 | 319.68 | 480.58 | 395.05 |
| High Peak and Dales | 318.82 | 123.29 | 343.09 | 179.89 |
| Hillingdon | 271.83 | 141.76 | 567.50 | 197.11 |
| Hinckley and Bosworth | 266.76 | 119.12 | 288.29 | 159.64 |
| Horsham and Chanctonbury | 314.82 | 92.26 | 182.17 | 146.45 |
| Hounslow | 301.84 | 174.07 | 499.87 | 217.57 |
| Huddersfield Central | 198.17 | 130.36 | 226.91 | 151.62 |
| Huntingdonshire | 329.18 | 136.40 | 542.41 | 202.69 |
| Hyndburn and Ribble Valley | 399.64 | 203.29 | 592.66 | 275.44 |
| Ipswich | 449.79 | 170.74 | 583.17 | 261.67 |
| Isle of Wight | 335.42 | 133.84 | 322.6 | 192.12 |
| Islington | 328.77 | 240.30 | 645.98 | 274.75 |
| Kennet and North Wiltshire | 230.09 | 112.08 | 393.20 | 157.01 |
| Kensington and Chelsea | 213.76 | 136.94 | 486.17 | 169.27 |
| Kingston | 347.98 | 123.79 | 599.61 | 199.63 |
| Knowsley | 411.39 | 363.37 | 650.26 | 390.80 |
| Lambeth | 282.05 | 227.97 | 673.31 | 256.98 |
| Langbaurgh | 355.58 | 250.69 | 326.71 | 277.59 |
| Leeds North East | 220.93 | 165.81 | 533.19 | 208.81 |
| Leeds North West | 303.88 | 230.74 | 558.31 | 265.64 |
| Leeds West | 513.90 | 232.46 | 525.30 | 311.22 |
| Leicester City West | 516.97 | 259.71 | 487.20 | 335.02 |
| Lewisham | 270.02 | 176.37 | 598.58 | 218.54 |
| Lincolnshire South West Teaching | 398.30 | 185.82 | 490.43 | 253.13 |
| Luton | 258.52 | 132.16 | 319.59 | 171.39 |
| Maidstone Weald | 304.92 | 109.13 | 368.45 | 167.31 |
| Maldon and South Chelmsford | 245.67 | 135.93 | 364.23 | 173.42 |
| Mansfield District | 350.79 | 263.79 | 363.34 | 289.26 |
| Medway | 323.16 | 152.35 | 426.22 | 206.00 |
| Melton, Rutland and Harborough | 225.32 | 105.37 | 394.68 | 151.16 |
| Mendip | 252.88 | 125.79 | 284.93 | 165.74 |
| Mid Devon | 278.66 | 173.12 | 569.97 | 229.25 |
| Middlesbrough | 582.39 | 431.12 | 550.58 | 472.49 |
| Mid-Hampshire | 533.77 | 130.55 | 256.26 | 219.13 |
| Mid-Sussex | 194.52 | 82.44 | 310.53 | 123.62 |
| Milton Keynes | 316.97 | 132.11 | 285.35 | 181.7 |
| Morecambe Bay | 409.92 | 212.73 | 543.11 | 279.37 |
| New Forest | 538.73 | 129.35 | 272.33 | 220.28 |
| Newark and Sherwood | 348.58 | 208.07 | 312.90 | 244.65 |
| Newbury and Community | 192.18 | 126.29 | 193.24 | 144.29 |
| Newcastle | 400.44 | 195.13 | 386.16 | 248.14 |
| Newcastle-under-Lyme | 256.90 | 129.31 | 120.55 | 151.97 |
| Newham | 222.43 | 199.10 | 378.25 | 212.39 |
| North and East Cornwall | 272.02 | 161.60 | 430.89 | 207.94 |
| North Birmingham | 317.64 | 117.72 | 338.01 | 178.44 |
| North Bradford | 421.89 | 153.78 | 316.22 | 221.63 |
| North Devon | 240.45 | 152.76 | 370.94 | 190.48 |
| North Dorset | 312.65 | 166.67 | 435.45 | 223.27 |
| North East Lincolnshire | 440.93 | 319.15 | 449.38 | 356.04 |
| North East Oxfordshire | 190.68 | 113.90 | 184.29 | 134.14 |
| North Eastern Derbyshire | 277.34 | 157.10 | 399.54 | 199.76 |
| North Hampshire | 356.83 | 156.87 | 357.49 | 211.03 |
| North Hertfordshire and Stevenage | 275.71 | 176.80 | 399.00 | 213.78 |
| North Kirklees | 316.55 | 173.11 | 278.63 | 213.26 |
| North Lincolnshire | 393.91 | 157.08 | 432.49 | 226.30 |
| North Liverpool | 443.38 | 419.25 | 844.28 | 450.54 |
| North Manchester | 881.71 | 307.90 | 414.89 | 444.86 |
| North Norfolk | 188.37 | 140.55 | 457.65 | 187.03 |
| North Peterborough | 509.36 | 338.08 | 770.53 | 405.15 |
| North Sheffield | 270.49 | 230.98 | 507.79 | 260.03 |
| North Somerset | 145.26 | 135.69 | 322.01 | 155.92 |
| North Stoke | 315.92 | 123.96 | 141.43 | 163.79 |
| North Surrey | 181.40 | 107.31 | 223.69 | 130.72 |
| North Tees | 509.27 | 284.49 | 493.31 | 345.28 |
| North Tyneside | 391.53 | 166.22 | 396.03 | 228.53 |
| North Warwickshire | 275.23 | 123.81 | 176.26 | 158.57 |
| Northampton | 455.47 | 177.79 | 257.53 | 240.73 |
| Northamptonshire Heartlands | 244.78 | 137.02 | 249.94 | 167.91 |
| Northumberland Care Trust | 326.40 | 182.04 | 459.24 | 231.45 |
| Norwich | 369.46 | 221.52 | 348.86 | 258.33 |
| Nottingham City | 512.61 | 272.23 | 414.55 | 329.60 |
| Oldbury and Smethwick | 373.61 | 194.22 | 524.86 | 260.42 |
| Oldham | 604.10 | 389.25 | 613.12 | 453.81 |
| Oxford City | 259.13 | 117.44 | 216.39 | 147.33 |
| Plymouth | 380.66 | 248.38 | 436.35 | 288.72 |
| Poole | 442.07 | 167.51 | 474.23 | 249.15 |
| Portsmouth City | 381.73 | 252.16 | 931.51 | 331.55 |
| Preston | 577.38 | 478.32 | 611.94 | 507.96 |
| Reading | 287.53 | 152.33 | 264.48 | 185.55 |
| Redbridge | 210.23 | 103.02 | 321.92 | 141.03 |
| Redditch and Bromsgrove | 323.51 | 116.75 | 297.81 | 171.22 |
| Richmond and Twickenham | 268.46 | 130.15 | 403.65 | 175.82 |
| Rochdale | 405.50 | 191.53 | 207.17 | 242.28 |
| Rotherham | 354.20 | 214.21 | 647.18 | 274.40 |
| Rowley Regis and Tipton | 426.07 | 175.14 | 360.52 | 243.62 |
| Royston, Buntingford and Bishop's St’ | 240.37 | 104.69 | 469.09 | 155.83 |
| Rugby | 249.35 | 157.07 | 265.57 | 184.10 |
| Rushcliffe | 355.53 | 136.52 | 318.94 | 193.18 |
| Salford | 556.93 | 275.46 | 386.57 | 341.50 |
| Scarborough, Whitby and Ryedale | 336.19 | 261.44 | 549.69 | 304.87 |
| Sedgefield | 353.72 | 183.22 | 486.96 | 239.66 |
| Selby and York | 337.47 | 129.04 | 265.71 | 178.37 |
| Sheffield South West | 218.46 | 127.53 | 438.92 | 170.87 |
| Sheffield West | 246.32 | 136.42 | 511.97 | 180.81 |
| Shepway | 249.39 | 171.83 | 358.57 | 206.24 |
| Shropshire County | 285.13 | 168.24 | 336.18 | 204.82 |
| Slough | 327.18 | 182.05 | 288.74 | 220.04 |
| Solihull | 332.46 | 143.22 | 445.29 | 206.00 |
| Somerset Coast | 238.37 | 148.87 | 353.26 | 186.58 |
| South and East Dorset | 339.45 | 118.30 | 295.65 | 179.21 |
| South Birmingham | 393.36 | 183.84 | 405.66 | 244.47 |
| South Cambridgeshire | 266.36 | 159.74 | 713.21 | 220.37 |
| South East Hertfordshire | 195.73 | 111.05 | 369.82 | 144.68 |
| South East Oxfordshire | 238.20 | 105.12 | 257.17 | 143.47 |
| South East Sheffield | 354.58 | 184.48 | 359.58 | 233.85 |
| South Gloucestershire | 211.60 | 100.52 | 238.23 | 132.31 |
| South Hams and West Devon | 204.79 | 131.28 | 490.41 | 181.40 |
| South Huddersfield | 161.58 | 134.15 | 209.10 | 144.85 |
| South Leeds | 476.28 | 269.85 | 473.98 | 328.09 |
| South Leicestershire | 336.35 | 154.09 | 369.04 | 205.76 |
| South Liverpool | 395.04 | 287.83 | 983.68 | 368.05 |
| South Manchester | 390.25 | 250.32 | 230.60 | 277.50 |
| South Peterborough | 365.04 | 226.58 | 360.04 | 264.18 |
| South Sefton | 336.16 | 255.58 | 707.55 | 306.04 |
| South Somerset | 309.22 | 136.30 | 369.39 | 192.51 |
| South Stoke | 366.91 | 157.82 | 108.57 | 195.23 |
| South Tyneside | 322.07 | 259.82 | 386.22 | 282.75 |
| South Warwickshire | 382.66 | 206.99 | 590.84 | 270.83 |
| South West Dorset | 416.60 | 173.47 | 362.86 | 238.04 |
| South West Kent | 353.48 | 148.03 | 679.45 | 234.64 |
| South West Oxfordshire | 153.23 | 98.49 | 136.18 | 112.48 |
| South Western Staffordshire | 291.05 | 106.47 | 205.72 | 148.17 |
| South Wiltshire | 298.77 | 163.34 | 416.50 | 211.88 |
| South Worcestershire | 361.15 | 97.56 | 276.96 | 163.03 |
| Southampton City | 616.37 | 192.42 | 255.12 | 274.55 |
| Southend on Sea | 196.16 | 111.82 | 293.56 | 147.26 |
| Southern Norfolk | 273.43 | 115.20 | 263.94 | 159.00 |
| Southport and Formby | 279.28 | 224.80 | 420.81 | 255.78 |
| Southwark | 257.76 | 210.37 | 819.18 | 249.12 |
| St Helens | 398.94 | 286.26 | 581.44 | 329.67 |
| St. Albans and Harpenden | 215.01 | 112.26 | 334.75 | 148.83 |
| Staffordshire Moorlands | 213.80 | 118.00 | 100.97 | 134.77 |
| Stockport | 346.03 | 180.17 | 328.51 | 225.29 |
| Suffolk Coastal | 163.39 | 123.25 | 506.45 | 171.74 |
| Suffolk West | 239.40 | 145.68 | 574.30 | 198.11 |
| Sunderland Teaching | 553.25 | 355.43 | 468.26 | 402.41 |
| Sussex Downs and Weald | 239.40 | 96.25 | 436.77 | 156.29 |
| Sutton and Merton | 378.58 | 132.14 | 225.82 | 188.21 |
| Swale | 358.83 | 161.64 | 445.36 | 223.43 |
| Swindon | 308.62 | 111.47 | 173.90 | 156.80 |
| Tameside and Glossop | 526.93 | 244.67 | 285.61 | 307.98 |
| Taunton Deane | 411.82 | 170.12 | 431.26 | 241.45 |
| Teignbridge | 305.58 | 162.52 | 550.14 | 233.24 |
| Telford and Wrekin | 419.22 | 213.61 | 363.80 | 267.80 |
| Tendring | 176.49 | 91.32 | 194.74 | 120.18 |
| Thurrock | 213.19 | 116.51 | 275.74 | 147.42 |
| Torbay | 291.58 | 228.94 | 560.10 | 279.86 |
| Tower Hamlets | 338.60 | 167.33 | 359.62 | 214.17 |
| Trafford North | 300.40 | 194.52 | 352.44 | 228.00 |
| Trafford South | 242.40 | 153.21 | 289.14 | 181.81 |
| Uttlesford | 197.93 | 116.34 | 311.28 | 147.13 |
| Vale of Aylesbury | 289.92 | 127.03 | 263.40 | 170.17 |
| Wakefield West | 414.94 | 139.80 | 356.22 | 209.47 |
| Walsall | 419.42 | 209.28 | 460.67 | 272.58 |
| Walthamstow, Leyton and Leytonstone | 183.49 | 173.58 | 598.64 | 195.58 |
| Wandsworth | 367.28 | 152.95 | 686.33 | 215.07 |
| Warrington | 479.77 | 308.51 | 859.60 | 379.42 |
| Watford and Three Rivers | 277.61 | 103.15 | 263.52 | 150.70 |
| Waveney | 290.04 | 157.38 | 320.42 | 200.50 |
| Wednesbury and West Bromwich | 408.22 | 218.02 | 463.75 | 277.34 |
| Welwyn Hatfield | 187.76 | 138.90 | 370.56 | 167.09 |
| West Cumbria | 463.37 | 279.59 | 582.17 | 337.99 |
| West Gloucestershire | 342.95 | 174.65 | 292.36 | 218.75 |
| West Hull | 359.15 | 311.41 | 456.52 | 331.77 |
| West Lancashire | 385.94 | 210.17 | 501.97 | 265.74 |
| West Lincolnshire | 412.90 | 254.60 | 659.60 | 318.64 |
| West Norfolk | 356.39 | 201.10 | 478.53 | 257.10 |
| West of Cornwall | 515.57 | 335.30 | 707.83 | 404.61 |
| West Wiltshire | 244.28 | 107.00 | 354.00 | 155.73 |
| Western Sussex | 314.56 | 121.09 | 280.36 | 174.07 |
| Westminster | 318.71 | 187.74 | 745.04 | 237.19 |
| Windsor, Ascot and Maidenhead | 287.30 | 106.85 | 282.40 | 154.00 |
| Witham, Braintree and Halstead | 284.82 | 104.45 | 430.89 | 165.90 |
| Woking Area | 296.39 | 127.10 | 395.14 | 178.79 |
| Wokingham | 193.50 | 91.82 | 223.24 | 119.82 |
| Wolverhampton City | 357.57 | 189.79 | 173.21 | 223.60 |
| Wycombe | 357.81 | 162.66 | 284.33 | 211.14 |
| Wyre | 329.51 | 178.94 | 372.49 | 227.23 |
| Wyre Forest | 271.64 | 121.57 | 259.52 | 160.97 |
| Yorkshire Wolds and Coast | 273.63 | 169.27 | 426.72 | 211.79 |
